# Educational Impact of a Pre-clinical Anesthesiology Elective

**DOI:** 10.7759/cureus.112636

**Published:** 2026-07-14

**Authors:** Suman M Atluri, Ali Khalifa

**Affiliations:** 1 Department of Anesthesiology, Baylor College of Medicine, Houston, USA

**Keywords:** anesthesiology education, curriculum development, educational outcomes, medical student electives, pre-clinical curriculum, undergraduate medical education

## Abstract

Anesthesiology is often underrepresented in pre-clinical curricula, leaving many students without structured exposure before clinical rotations. This study evaluated an eight-week pre-clinical anesthesiology elective at Baylor College of Medicine, in which first-year medical students voluntarily attended weekly lectures from anesthesiology faculty across four affiliated hospitals covering anesthetic history, modern anesthetic delivery, regional and obstetric anesthesia, and ethics. Anonymous pre- and post-course surveys (13 items, 1-10 scale) assessed specialty interest and self-reported understanding; only the 12 students completing both surveys were included in the analysis, performed via paired t-tests. Self-reported understanding improved significantly across all knowledge domains, including history of anesthesia, early anesthetic techniques, ether/nitrous oxide use, wartime anesthesia, modern techniques and pharmaceuticals, regional/local anesthesia, obstetric anesthesia, and ethical issues (all p < 0.05, most p < 0.001), with the largest gains observed in comfort discussing anesthesiology content and clinically relevant techniques. Interest in anesthesiology, surgical specialties, and non-surgical specialties did not change significantly, likely reflecting high baseline interest among self-selected, voluntary participants. These preliminary findings suggest that even brief pre-clinical exposure to anesthesiology may improve self-perceived understanding and confidence independent of shifts in specialty interest. Given the small sample and reliance on self-reported outcomes, larger studies are needed to confirm these findings.

## Introduction

The practice of anesthesiology draws heavily on foundational concepts in physiology, pharmacology, and team-based care, offering medical students an opportunity to apply and reinforce pre-clinical coursework in a clinically relevant context, regardless of eventual specialty choice [1]. However, representation of anesthesiology in pre-clinical curricula remains variable across institutions, and many students begin clinical training without structured instruction in anesthesia-related concepts [2].

Inclusion of this content early in medical education may help students better understand the specialty and prepare for subsequent clinical rotations [3]. Prior studies suggest that specialty-specific pre-clinical electives can enhance medical students' learning experiences [4]; however, such courses are not uniformly available across medical schools, and the existing literature remains limited, particularly with respect to whether brief, voluntary pre-clinical electives can produce measurable gains in students' subject-specific understanding and confidence, as distinct from shifts in specialty interest. We hypothesized that participation in a pre-clinical anesthesiology elective would be associated with significant increases in students' self-reported understanding of and confidence in anesthesia-related content, but would have a more limited effect on specialty interest, given that course enrollment was voluntary and likely enriched for students with pre-existing interest in the field. We evaluated the educational impact of a pre-clinical anesthesiology elective at Baylor College of Medicine on medical students' interest, perceived understanding, and confidence across multiple anesthesia-related domains.

## Technical report

Methods

An eight-week elective focused on the history and clinical foundations of anesthesiology was offered to first-year medical students during the academic year. The course consisted of weekly one-hour lectures delivered by anesthesiology faculty representing a broad range of subspecialty backgrounds and clinical practice settings across four affiliated hospitals within the institution. Course content included early anesthetic techniques, modern anesthetic delivery, regional anesthesia, obstetric anesthesia, pain management, and ethical considerations in anesthesiology. Enrollment in the class was voluntary. Of 55 first-year medical students enrolled in the elective, 12 (21.8%) completed both the pre-course and post-course surveys and were included in the final analysis. Students were invited to complete confidential pre-course and post-course surveys administered electronically through Google Forms (Google LLC, Mountain View, CA, USA); responses were linked using students' institutional ID numbers to permit pre-post pairing, and these identifiers were removed prior to analysis. The survey instrument was developed by the course directors specifically for this elective and has not been independently validated; it is provided in the Appendices. The instrument was not pilot-tested prior to administration, and no formal assessment of content validity, test-retest reliability, or internal consistency was conducted. The survey consisted of 13 items rated on a 1-10 scale, assessing interest in anesthesiology and other specialties, as well as self-reported understanding of anesthesia-specific topics. Only students who completed both the pre- and post-course surveys were included in the analysis.

Attendance was tracked at every session, and students were required to attend a minimum of seven of the eight weekly lectures to pass the elective; no students joined after the course began, and no student who attended fewer than seven sessions was included in this analysis. Although completion of the pre- and post-course surveys was not mandatory, survey non-completion was largely attributable to students not submitting a pre-course survey at all or submitting a pre-course survey but not the corresponding post-course survey, rendering their responses unusable for paired analysis.

Pre- and post-course survey responses were compared using two-tailed paired t-tests. Descriptive statistics are reported as mean ± standard deviation, and statistical significance was defined as p < 0.05. For each item, we report the t statistic, degrees of freedom, exact p-value, mean difference with 95% confidence interval, and effect size (Cohen's dz for paired samples). Analyses were performed using Microsoft Excel (Microsoft Corp., Redmond, WA, USA). Because survey items assessed distinct educational domains, no formal adjustment was made for multiple comparisons. Normality of difference scores was not formally assessed; results should therefore be interpreted with appropriate caution.

Results

Of the 55 students enrolled, 12 (21.8%) completed both the pre-course and post-course surveys and were included in the final analysis; the remaining 43 enrolled students did not complete one or both surveys and were excluded. Students demonstrated statistically significant increases in self-reported understanding across multiple anesthesia-related topics. These included orientation to anesthesia as a medical specialty, ability to discuss the history of anesthesia, and knowledge of early anesthetic techniques, ether and nitrous oxide use, anesthesia during wartime, modern anesthetic techniques and pharmaceuticals, regional and local anesthesia, obstetric anesthesia and pain management, and ethical issues in anesthesiology (all p < 0.05; full test statistics, 95% confidence intervals, and effect sizes are reported in Table 1). The magnitude of improvement was greatest for items related to the history of anesthesia and early anesthetic techniques (Cohen's dz > 3.0), reflecting very large effect sizes for these content areas. In contrast, there were no statistically significant changes in students' self-reported interest in anesthesiology as a specialty, interest in surgical specialties, or interest in non-surgical specialties following completion of the elective. A summary of pre- and post-course survey results is shown in Table 1.

**Table 1:** Pre- and Post-course Survey Responses for a Pre-clinical Anesthesiology Elective, With Paired T-test Statistics, 95% Confidence Intervals, and Effect Sizes (n = 12) Values are reported as mean ± standard deviation. Survey items were rated on a 1-10 scale. Pre- and post-course responses were compared using two-tailed paired t-tests (degrees of freedom = 11 for all comparisons). Mean differences are reported with 95% confidence intervals, and effect sizes are reported as Cohen’s dz for paired samples. Statistical significance was defined as p < 0.05 and denoted with an asterisk (*).

Survey Item	Pre-course (Mean ± SD)	Post-course (Mean ± SD)	Mean Difference (95% CI)	t(11)	Exact p-value	Cohen's dz
Interest in anesthesiology as a specialty	6.92 ± 1.93	7.42 ± 1.56	0.50 (-0.38 to 1.38)	1.25	0.236	0.36
Interest in a surgical specialty	6.42 ± 2.71	6.42 ± 3.00	0.00 (-0.66 to 0.66)	0.00	1.000	0.00
Interest in a non-surgical specialty	6.33 ± 1.50	6.33 ± 2.10	0.00 (-0.90 to 0.90)	0.00	1.000	0.00
Knowledge of anesthesia as a medical specialty	4.92 ± 1.68	6.50 ± 1.45	1.58 (0.30 to 2.87)	2.71	0.020*	0.78
History of anesthesia	1.75 ± 1.14	6.50 ± 1.31	4.75 (3.81 to 5.69)	11.08	<0.001*	3.20
Understanding of the everyday work life of an anesthesiologist	4.50 ± 1.24	6.33 ± 2.23	1.83 (0.38 to 3.29)	2.77	0.018*	0.80
Early anesthetic techniques	2.00 ± 1.21	6.83 ± 1.47	4.83 (3.83 to 5.84)	10.56	<0.001*	3.05
Ether and nitrous oxide use	2.33 ± 1.67	7.17 ± 1.64	4.83 (3.63 to 6.04)	8.82	<0.001*	2.55
Anesthesia use during wartime	2.17 ± 2.04	6.50 ± 1.68	4.33 (2.43 to 6.24)	5.01	<0.001*	1.45
Modern anesthetic techniques and pharmaceuticals	2.50 ± 1.68	7.00 ± 1.65	4.50 (3.13 to 5.87)	7.24	<0.001*	2.09
Regional and local anesthesia	3.17 ± 2.59	6.83 ± 1.70	3.67 (1.92 to 5.41)	4.63	<0.001*	1.34
Obstetric anesthesia and pain management	3.00 ± 1.91	7.08 ± 1.56	4.08 (2.69 to 5.48)	6.45	<0.001*	1.86
Ethical issues in anesthesiology	3.75 ± 2.70	6.92 ± 1.68	3.17 (1.99 to 4.34)	5.93	<0.001*	1.71

## Discussion

These findings, which align with prior studies of specialty-specific pre-clinical electives, suggest that the primary effect of the course was on students' engagement with concepts relevant to anesthesia rather than on short-term shifts in specialty preference [5]. The absence of significant changes in interest-related survey items should be interpreted in the context of both baseline scores and course enrollment characteristics. Students entered the elective with relatively high baseline interest in anesthesiology and surgical specialties, which perhaps limited the degree of change detectable on interest-related survey items. Further, enrollment in the class was voluntary, and participants may have had a preexisting interest in anesthesiology or perioperative medicine. Accordingly, stable interest scores likely reflect reinforcement of existing interest rather than a lack of educational effect.

These results are broadly consistent with prior reports of preclinical anesthesiology and surgery electives. Walsh et al. similarly found that a preclinical anesthesiology elective improved medical students' knowledge and attitudes without necessarily shifting specialty preference [3], and Drolet et al. reported that a mentorship-based preclinical surgery elective increased exposure and confidence among participating students [4]. Our findings extend this literature by quantifying large, item-level gains in self-reported understanding (Cohen's dz ranging from 0.78 to 3.20) across a broad range of anesthesia-specific content areas, suggesting that even a brief, lecture-based pre-clinical elective can produce substantial gains in self-perceived understanding. Similarly, Cantwell et al. found that a pre-clinical emergency medicine milestones elective produced significant gains in students' self-reported preparedness without a significant change in specialty interest, closely mirroring the pattern observed in our study [6]. A pilot study of a child and adolescent psychiatry elective similarly reported large effect sizes for gains in students' self-reported comfort and confidence, alongside only a small, non-significant increase in specialty interest, suggesting this dissociation between knowledge/confidence gains and interest shifts may be a general feature of brief specialty-specific pre-clinical electives [7]. Generalizability of these findings is limited by the single-institution, voluntary-enrollment design: students who elect to take such a course are likely not representative of the broader medical student population, and the 21.8% response rate among enrolled students raises the possibility of response bias, as students who completed both surveys may have differed systematically from those who did not. Replication at other institutions, with larger and more representative samples and higher survey completion rates, would help clarify how broadly these findings apply.

Limitations of this study included a small sample size, a low survey response rate (12 of 55 enrolled students, 21.8%), and voluntary survey participation, all of which limit the precision and generalizability of these findings. We were unable to compare survey completers to non-completers on observable characteristics such as attendance records or course engagement, which limits our ability to assess the direction or magnitude of any resulting selection bias. Critically, all outcomes were based on students' self-reported perceptions of their own understanding and confidence rather than on objective assessments of knowledge, such as examination performance; self-report measures are subject to social desirability and recall bias and may not correspond to actual gains in competency. Long-term outcomes such as performance in clinical clerkships or residency application choices were not assessed, and the survey instrument itself was developed for this course and has not been validated against an external standard. Despite these limitations, the pattern of improvements suggests that even brief pre-clinical exposure to anesthesiology can yield meaningful perceived benefits. Further work using validated instruments and objective measures of knowledge is needed to confirm and extend these preliminary findings.

## Conclusions

In conclusion, these preliminary findings suggest a potential value of a pre-clinical anesthesiology elective in improving students' self-perceived understanding of and confidence in anesthesia-related content, independent of changes in specialty interest. The course provided structured instruction in areas commonly introduced later in medical training. Given the small sample size, low response rate, and reliance on an unvalidated, self-reported survey instrument, these results should be interpreted as hypothesis-generating rather than confirmatory. Larger, multi-institutional studies using validated instruments and objective measures of knowledge are needed before firm conclusions can be drawn about the role of specialty-specific electives early in medical education.

## Appendices

History of anesthesia: beginning/end of course survey

Each item was rated on a 10-point Likert scale (1 = lowest, 10 = highest), administered identically at the beginning and end of the course.

What is your current level of interest in anesthesiology as a specialty? (1 = Not at all interested, 10 = Very interested)

What is your current level of interest in a surgical specialty? (1 = Not at all interested in surgery, 10 = Very interested in surgery)

What is your current level of interest in a non-surgical specialty? (1 = Not at all interested, 10 = Very interested)

What is your current level of understanding of anesthesia as a medical specialty? (1 = Not very familiar, 10 = Very familiar)

What is your current level of confidence in being able to discuss the history of anesthesia with physicians? (1 = Not confident, 10 = Very confident)

What is your current level of understanding in the everyday work life of an anesthesiologist? (1 = Not familiar, 10 = Very familiar)

What is your current level of understanding of early anesthetic techniques? (1 = Not familiar, 10 = Very familiar)

What is your current level of understanding in ether and nitrous oxide use as anesthetics? (1 = Not familiar, 10 = Very familiar)

What is your current level of understanding in anesthesia use during war time? (1 = Not familiar, 10 = Very familiar)

What is your current level of understanding in modern anesthesia techniques and pharmaceuticals? (1 = Not familiar, 10 = Very familiar)

What is your current level of understanding in regional and local anesthesia? (1 = Not familiar, 10 = Very familiar)

What is your current level of understanding in anesthesia in obstetrics and pain management? (1 = Not familiar, 10 = Very familiar)

What is your current level of understanding in ethical issues that could face the field of anesthesia? (1 = Not familiar, 10 = Very familiar)
